# Monitoring mobility in older adults using a Global Positioning System (GPS) smartwatch and accelerometer: A validation study

**DOI:** 10.1371/journal.pone.0296159

**Published:** 2023-12-21

**Authors:** Marla Beauchamp, Renata Kirkwood, Cody Cooper, Matthew Brown, K. Bruce Newbold, Darren Scott, on behalf of the MacM3 team

**Affiliations:** 1 School of Rehabilitation Science, McMaster University, Hamilton, Ontario, Canada; 2 School of Earth, Environment & Society, McMaster University, Hamilton, Ontario, Canada; Helwan University Faculty of Engineering, EGYPT

## Abstract

There is growing interest in identifying valid and reliable methods for detecting early mobility limitations in aging populations. A multi-sensor approach that combines accelerometry with Global Positioning System (GPS) devices could provide valuable insights into late-life mobility decline; however, this innovative approach requires more investigation. We conducted a series of two experiments with 25 older participants (66.2±8.5 years) to determine the validity of a GPS enabled smartwatch (TicWatch S2 and Pro 3 Ultra GPS) and separate accelerometer (ActiGraph wGT3X-BT) to collect movement, navigation and body posture data relevant to mobility. In experiment 1, participants wore the TicWatchS2 and ActiGraph simultaneously on the wrist for 3 days. In experiment 2, participants wore the TicWatch Pro 2 Ultra GPS on the wrist and ActiGraph on the thigh for 3 days. In both experiments participants also carried a Qstarz data logger for trips outside the home. The TicWatch Pro 3 Ultra GPS performed better than the S2 model and was similar to the Qstarz in all tested trip-related measures, and it was able to estimate both passive and active trip modes. Both models showed similar results to the gold standard Qstarz in life-space-related measures. The TicWatch S2 demonstrated good to excellent overall agreement with the ActiGraph algorithms for the time spent in sedentary and non-sedentary activities, with 84% and 87% agreement rates, respectively. Under controlled conditions, the TicWatch Pro 3 Ultra GPS consistently measured step count in line with the participants’ self-reported data, with a bias of 0.4 steps. The thigh-worn ActiGraph algorithm accurately classified sitting and lying postures (97%) and standing postures (90%). Our multi-sensor approach to monitoring mobility has the potential to capture both accelerometer-derived movement data and trip/life-space data only available through GPS. In this study, we found that the TicWatch models were valid devices for capturing GPS and raw accelerometer data, making them useful tools for assessing real-life mobility in older adults.

## Introduction

Mobility problems are an early predictor of disability [1], hospitalization [2] and death [3]. Recent studies suggest that the first change in functioning in older adulthood is often an “asymptomatic” decline in mobility, referred to as preclinical mobility limitation (PCML) [4]. At the stage of PCML an older person may be able to compensate for underlying deficits or disease by modifying their task performance without a strong perception of difficulty (e.g., using the handrail to climb the stairs) [5, 6]. Older adults with PCML are at higher risk for progression to major mobility disability [7, 8]. Accordingly, it is important to determine the most valid and reliable method of identifying individuals at risk for PCML, to mitigate and prevent progression to mobility disability.

Mobility is usually assessed with self-reported measures or through the performance of mobility-related tasks [9, 10]. However, both types of measures are obtained at discrete times and may fail to represent the actual mobility of the individual in everyday life [11]. The challenge, therefore, is how to ascertain the “real-life mobility” of community-dwelling older adults. With advances in technology, device-based measures allow for continuous, objective, and unobtrusive assessment of mobility both in and out-of-home. Modern wearable devices (i.e., smartwatches) are often equipped with a variety of sensors including built-in accelerometers, gyroscopes, inclinometers, and satellite navigation (i.e., Global Positioning System (GPS) receiver) that can provide information such as body position [12], step counts [13] and life-space mobility [14]. Such measures can inform about both sedentary behavior and physical activity levels, which have been associated with adverse health outcomes, including reduced physical function [15, 16]. Together, accelerometry and GPS allow for the estimation of the quantity and quality of everyday activities that could help identify early declines in mobility. However, few studies to date have used both technologies simultaneously to comprehensively measure real-life mobility [17], with much of the work to date requiring participants to carry a second device to collect the GPS information

Of the available devices, wrist-worn activity monitors have become increasingly popular and are well-accepted among older adults, thereby improving study compliance [13, 18, 19]. These smartwatches have been validated for physical activity energy expenditure and have been successfully used in large cohort studies [13]. However, despite the growing number of studies on wearable sensors in older adults, most of them focus only on measuring step counts and physical activity intensity, neglecting important information on body posture and life-space mobility. Importantly, GPS technology can provide a more detailed understanding of mobility patterns and environmental influences than previously possible.

In 2018, we initiated the McMaster Monitoring My Mobility project (MacM3), a research platform at McMaster University focused on real-life mobility monitoring using multi-sensor wearable technology. The MacM3 cohort study aims to collect comprehensive mobility data (accelerometry and GPS) every 4 months for up to 2 years in 1,500 adults 65 years and older, who have no mobility limitations or have pre-clinical mobility limitations at baseline [20]. Prior to this validation study, the MacM3 technical team evaluated numerous commercially available wearable devices to determine the most suitable solution for collecting both movement and navigation data in a large cohort. The TicWatch models S2 and Pro 3 Ultra GPS (www.mobvoi.com) were ultimately selected for this validation study because it has a standalone GPS with long battery life, a Google Wear OS platform for customized data collection, and can collect raw data from other sensors independently. This eliminates the need for a secondary device and allows continuous location tracking. A second device, the medical-grade ActiGraph wGT3X-BT worn on the thigh, was selected to measure body posture. This multi-sensor approach (smartwatch on the wrist and thigh-worn device) was also approved by our stakeholder advisory committee consisting of older adults and caregivers.

The overall objective of this study was to validate our multi-sensor approach for monitoring real-life mobility in older adults. We conducted two experiments to validate the TicWatch for collecting movement and navigation data, while also testing the body posture algorithms from the ActiGraph wGT3X-BT. In experiment 1, we compared the TicWatch S2 against the Qstarz BT-Q1000X GPS Data Logger to measure life-space mobility, trip frequency and duration. Additionally, we assessed the agreement of the TicWatch S2 in measuring step count against participants’ self-reports, as well as activity counts per minute (CPM) and sedentary and non-sedentary activity against the ActiGraph’s proprietary algorithms. In experiment 2, we compared the TicWatch Pro 3 Ultra GPS against a stand-alone Qstarz BT-Q1000X GPS Data Logger to measure life-space mobility, trip frequency and duration, and mode of transportation. We evaluated the level of agreement between the TicWatch Pro 3 Ultra GPS in measuring steps compared to self-reported step counts that were tracked by the participants during the walking tests. Additionally, we tested two different GPS configurations of the TicWatch Pro 3 Ultra GPS in real-life settings to observe battery life performance. Finally, we evaluated the agreement of the thigh-worn algorithms of the ActiGraph wGT3X-BT with participants’ self-report in identifying body posture (lying and sitting from standing). We hypothesized that the TicWatch models would perform similarly to the gold standard GPS logger (Qstarz) and that the thigh-worn algorithms of the ActiGraph would have the highest accuracy for detecting body posture.

## Materials and methods

### Participants

A convenience sample of 25 participants was recruited primarily via word of mouth from January to November 2021. Volunteers were eligible for inclusion if they were 55 years or older and were able to walk independently with or without an assistive device (e.g., cane or rollator). At the time of consent, participants were asked to provide demographic and medical information, including age, gender, height, mass, and level of education. They also completed the Preclinical Mobility Limitation scale (PCML) [20] and answered questions about fall history and balance problems. The PCML assesses participants’ mobility over the past six months by asking their ability to walk 0.5 km, 2 km, and climbing a single flight of stairs. Participants indicating no difficulties are questioned about any task modifications and then classified as having: 1) no mobility limitation (no difficulty or modification), 2) early mobility limitation (one or more modifications), or 3) minor mobility limitation (some task difficulty). There were no exclusion criteria for participation in the study. This study was approved by the Hamilton Integrated Research Ethics Board (HiREB -2019-7610-GRA) and all participants provided informed verbal consent prior to participation.

### Measurement instruments

#### GPS data logger

The Qstarz BT-Q1000X GPS Data Logger is a portable standalone wireless device that uses GPS, a satellite-based navigation system, for logging geolocation data [21]. This device has been used extensively in previous large-population studies on older adult mobility due to its long battery life and spatial accuracy [21]. As noted by Duncan et al. [22], the location accuracy of the Qstarz is 3 meters (50% precision), the battery life is 42 hours, and it can store up to 400,000 points [22]. The Qstarz Data Logger uses signals from satellite constellations to determine position (longitudinal and latitudinal coordinates), altitude, and time. The device was set up to gather location coordinates at intervals of 5 seconds.

#### TicWatch models and custom software (Ivy and Clover)

The TicWatch S2 and the Pro 3 Ultra GPS are marketed as waterproof military-grade smartwatches designed by Mobvoi and use the Wear OS operating system by Google^TM^. Both watches have a built-in tri-axial accelerometer, gyroscope, heart rate sensor, and GNSS receiver. The watches were physically altered to prevent participant interaction by removing the action button. A custom data-collection application was developed to optimize data quality and battery life. Ivy is an app designed to run on any wearable device running Google Wear OS and to allow for continuous, non-intrusive, long-term measurements of key mobility-related parameters. It collects and stores data from the following onboard sensors: accelerometer, gyroscope, location (GPS), and heart rate. The app additionally collects information on step counts, current activity type predictions (using the Google Activity Recognition API), on-body detection, and device power/battery states. Ivy also provides a modular list of settings which control behavior of the TicWatch’s sensors, namely the frequency of accelerometry and GPS receiver to address battery constraints. Our second app, Clover, has been designed to support Ivy as a Windows companion app with a user-friendly graphical interface that automates the process of setting up Ivy on a smartwatch, downloading collected data, and preparing devices for distribution. Clover has the capability to conduct initial checks on data quality, including an assessment of the number of days the watch was worn.

The TicWatch models have an onboard GPS antenna that acts as a receiver for logging position coordinates. Data were stored on the watches and encrypted to ensure the privacy of information. The watch was tested extensively to determine ideal collection settings and configured to collect location coordinates every 20 seconds when Google’s Activity Recognition (GAR) detected an active motion state (e.g., walking, in-vehicle, etc.) with good satellite reception. The watch was configured to collect location data much less frequently when GAR detected a still activity state or had poor satellite reception. In accordance with previous studies, we considered 8 hours as the minimum GPS wear time threshold [23, 24].

Using Ivy, we controlled how GPS data were collected by the TicWatch. GPS data from the TicWatch S2 were collected at different intervals depending on the predicted activity state of the device using the GAR API to accommodate battery life. Due to the updated hardware of the TicWatch Pro 3 Ultra GPS, we chose to test two GPS configurations to observe battery life performance in a real-life mobility setting, both of which had a significantly higher collection frequency than the S2 model. The two GPS configurations tested were 1) periodic fix collection every 10 seconds (i.e., the GPS receiver turned off for 10 seconds before searching for a new location point), and 2) stay connected fix collection every 5 seconds (i.e., the GPS receiver never turns off, allowing for more continuous data collection). GPS data collection was programmed to stop collecting data at a low battery threshold because GPS has a substantially higher battery drain rate compared to the other sensors. This ensured that the other sensors could continue collecting for the whole study day, while still allowing several hours of GPS data capture. The battery threshold was set to 30% on the S2 and 16% on the Pro 3 Ultra. These thresholds were chosen by conducting several wear tests to assess non-GPS sensor battery drain rate. Notably, the more up-to-date hardware on the Pro 3 Ultra yielded more efficient battery rates in our tests, and thus the threshold was set at a lower percentage for that device.

Based on other literature on GPS-based indicators of daily mobility in older adults, the following metrics were examined: a) maximum distance from home [25], defined as the farthest planar/straight-line distance (in meters) from the participants’ home location that was travelled to; b) minimum Convex Hull (MCH) [14], defined as the smallest polygon in which no internal angle exceeds 180 degrees, and which contains all collected points (area and perimeter are reported in meters squared and meters, respectively); c) trip frequency, defined as the total number of trips taken per day [14, 26], and d) trip duration, defined as the total time duration of trips taken per day. A comprehensive description of the methodology used to define trips can be found in the “Data Cleaning and Processing” section.

We additionally measured the amount of time spent in active (i.e., non-motorized) and passive travel (i.e., motorized) modes in the second experiment due to the improved hardware capabilities of the TicWatch Pro 3 Ultra GPS

#### ActiGraph wGT3X-BT

The ActiGraph wGT3X-BT (ActiGraph, Pensacola, FL) is a medical-grade monitor device considered the gold standard in accelerometry movement analysis for research [27]. A built-in triaxial accelerometer captures high-resolution raw acceleration data, which is recorded continuously. We used the wrist-worn ActiGraph for validating the accelerometer on the TicWatch and evaluated the thigh-worn ActiGraph for measuring body posture in our cohort. Data were downloaded using the ActiLife software (version 6.11.4).

The following measures were obtained from the accelerometers: counts per minute (based on the frequency and intensity of the raw acceleration data—values are the sum of post-filtered accelerometer values into 60-epochs [28]), activity intensity classification (sedentary and non-sedentary) [29] and body posture (time spent in lying down and sitting versus standing).

### Protocol of experiments

#### Experiment 1

Participants were provided with three devices, the TicWatch S2, Qstarz GPS Data Logger and the ActiGraph. They were instructed to wear the TicWatch S2 and the ActiGraph on the non-dominant wrist simultaneously and to carry the GPS data logger with them whenever they travelled outside their homes. They were instructed to charge the TicWatch S2 and Qstarz every night using the chargers provided.

The ActiGraph has a long-lasting battery and did not require charging for the duration of the study. In the package, participants also received a booklet containing guidelines and instructions for using the devices and logging activity data, information on performing the step count tasks and a feedback questionnaire. Trip diary information included trips taken from home and returning home. To simplify the recording process for participants in their diaries and reduce the likelihood of reporting errors, we asked participants to record meaningful bouts of activities in terms of their body posture (e.g., sitting, standing or lying) or whether they were exercising as they were able to throughout the day. Participants were also asked to write the address or the intersection of the places they visited.

#### Experiment 2

Participants were provided with three devices, the TicWatch Pro 3 Ultra GPS, Qstarz GPS Data Logger and the ActiGraph. They were instructed to wear the TicWatch Pro 3 Ultra GPS on the non-dominant wrist, to carry the GPS whenever they travelled outside the home and to record the times they took the watch on and off. They were also instructed to attach the ActiGraph to the anterior aspect of the left or right thigh just above the kneecap using the adhesive patches provided to perform the body posture tasks described below. After the experiment, participants completed a short questionnaire aimed at gathering feedback on their experience wearing the thigh device. The questionnaire asked participants if they would be willing to wear the devices for 10 consecutive days. We also inquired about the clarity of instructions on how to attach the device to the thigh, whether it was easy for them to remember to use the devices regularly, and if the adhesive caused any redness or irritation. Additionally, we requested participants to indicate their preference for a specific adhesive patch.

**Fig 1:**
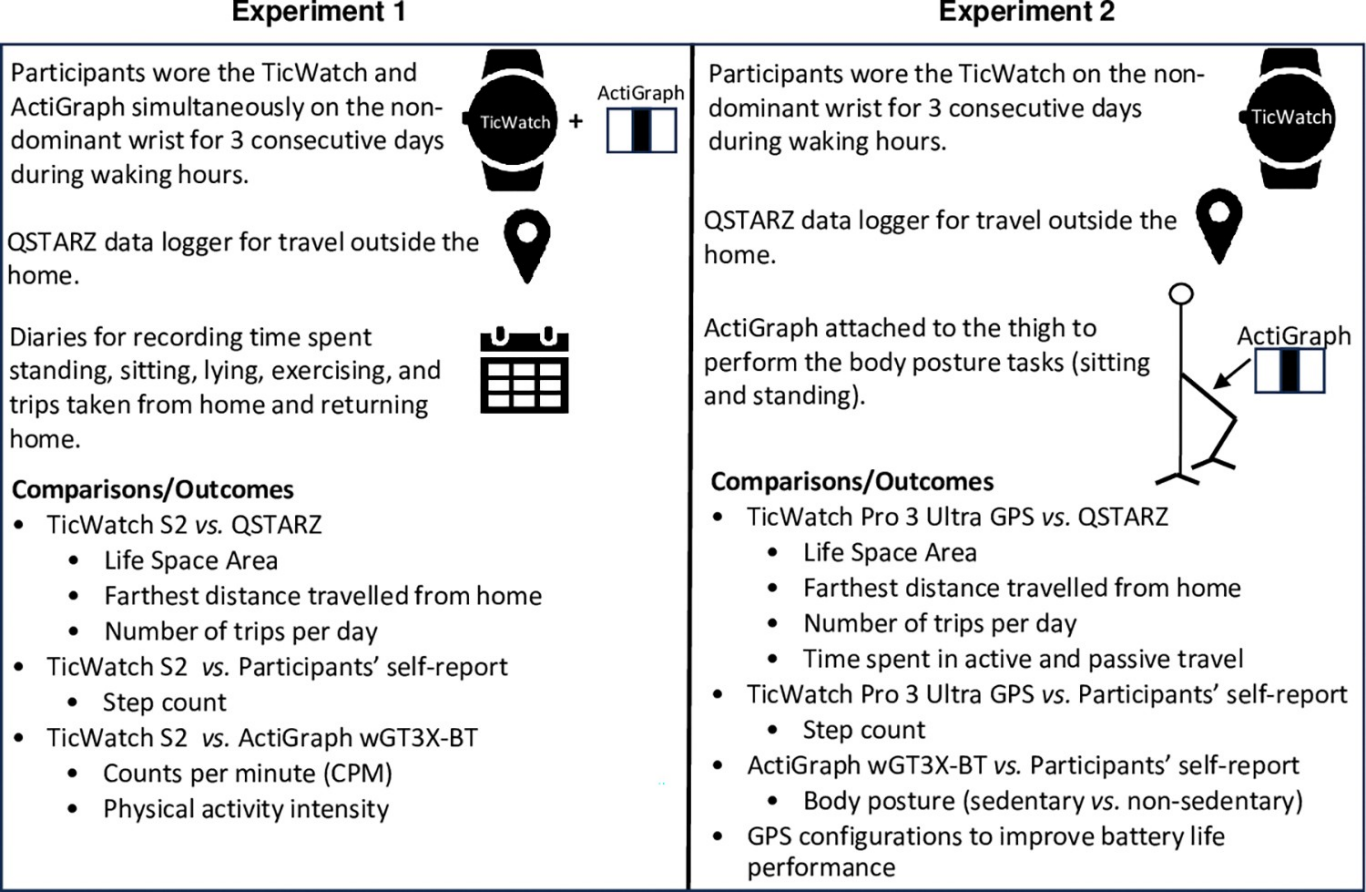
Observed variations between conducted experiments.

In both experiments, participants were asked to wear the devices for three consecutive days during waking hours and remove the devices for showering and water activity purposes. The devices and information sheet were returned by courier with a pre-paid box provided. Upon receipt of the devices, data were extracted for analysis. Fig 1 illustrates the variations within each experiment. Experiment 1 was conducted from January to June 2021 and experiment 2, from September to November 2021.

### Assessment of step count and body position

For step count, in both experiments, participants were instructed to remain still for about 30 seconds, then begin walking at their normal pace in a comfortable space while keeping track of their steps for 50 to 100 steps. At the end of the step count, they were asked to remain still for approximately 30 seconds. Participants recorded the start and end times, and their number of steps on the booklet provided. The step count task could be performed either at home or outdoors.

In Experiment 2, participants were instructed to perform two tasks: sit on the edge of a bed (or sofa) and stand still, with or without support, for 5 minutes in each activity. The first and last 45 seconds of data from each activity were discarded to avoid potential participant error in recording time and to exclude the transition times.

### Data cleaning and processing

The GPS data collected by the Qstarz and TicWatch models were first cleaned by excluding any points with speeds above 160 km/h, as the fastest roadways in our study area have a maximum speed limit of 110 km/h. We then processed each participant’s data to ensure the time periods compared between devices were identical, such that discrepancies due to battery life or participant error were excluded from the analysis. Measures related to the life-space area, such as maximum distance from home, minimum convex hull (MCH), and standard deviational ellipse (SDE), were calculated using ArcGIS® Pro, a desktop Geographic Information System application developed by Esri®. Each participant’s trip frequency and trip duration were determined using GPS data collected by both Qstarz and TicWatch. To accomplish this, we adapted the stop and trip detection algorithm of Montoliu et al. [30]. We chose to use algorithm settings proposed by Fillekes et al. [14] for trip detection and used them to derive trip frequency and duration independently for each device. With these algorithms, GPS data were classified into “stop” and “move” segments. Stops were defined as segments of GPS points in which a participant remained for at least 5 minutes, where the maximum distance a participant could move and still be considered stopped was 75 meters. This was determined using the median latitude/longitude of included GPS points, as well as their timestamps. If two consecutive GPS points labelled as stops had a data gap of greater than 60 minutes, they were not considered as part of the same stop event. Move segments were defined as GPS points in between identified stops that were longer than 3 minutes, with a minimum of 100 meters travelled between the farthest points in the segment. The algorithm identified trip times for the Qstarz data were manually verified on a case-by-case basis through an examination of GPS tracks to ensure accuracy. We then compared the algorithm-derived measures from the TicWatch against these results to evaluate the accuracy of the TicWatch in capturing trip information. Additionally, we used the method proposed for segmenting GPS segments into active (non-motorized) and passive (motorized) trips, which is adapted from the work of Carlson et al. [31] and Vanwolleghem et al. [32]. Specifically, trips with 90^th^ percentile speed ≥ 25 km/h were classified as passive, whereas trips below that threshold were classified as active.

Accelerometer data were collected at different frequencies for the ActiGraph (30Hz) and TicWatch S2 (25Hz) and Pro 3 Ultra GPS (50Hz). TicWatch data was resampled to match the ActiGraph’s frequency.

In Experiment 1, the accelerometer data from the ActiGraph and TicWatch S2 were screened for the period of wear times using the method described by Choi et al. [33]. We first determined the activity counts per minute (CPM) using a Python script that generates the ActiGraph physical activity counts [28]. We applied the script on both the S2 and ActiGraph devices. Based on the activity counts and using an epoch length of 60 seconds, non-wear time was defined as 90 consecutive minutes of zero counts, with an allowance of 2 minutes of nonzero counts, provided there were 30-minute consecutive zero counts before and after that allowance [33]. Wear times in Experiment 2 were obtained using the on-body sensor of the TicWatch Pro 3 Ultra GPS. The low-latency on-body sensor discerns whether the TicWatch is worn on the wrist or not, utilizing the heart rate monitoring sensors for this purpose. For the purpose of the experiments, a minimum of 8 hours per day of wear time was considered for data analysis.

To evaluate PA intensity, we computed the vector magnitude (VM) by taking the square root of the summed squared counts per minute for each axis on both devices. The VM counts were then calculated per 60-second epoch, and we applied the cut-off scores developed by Montoye et al. [29] specifically for wrist-worn devices. We classified activities as "sedentary" if the VM counts were below 2,860 and collapsed light and moderate/vigorous activity categories into "non-sedentary" which included VM counts of 2,860 or higher [29]. Following, we determined the time, in minutes, spent on both sedentary and non-sedentary behaviors based on participant-reported activities, such as exercise, sitting, lying, and walking. Additionally, we calculated the mean activity counts per 60-second epoch for these reported activities, analyzing data from both ActiGraph and TicWatch S2. To ensure an accurate comparison of accelerometer data, we restricted our analysis to the periods when participants reported wearing both the TicWatch and ActiGraph devices simultaneously.

Step count was obtained directly using the step count and step detector sensors from the TicWatch models. In Experiment 1, we selected the step counter that keeps track of the total number of steps taken over time. In Experiment 2, we used the step detector that detects when a step is taken and generates an event each time it detects a step, but it does not keep track of the total number of steps taken. Body posture classification in Experiment 2 was obtained using the thigh-worn algorithm from the ActiGraph that relies on movement and the thigh angle to accurately classify lying and sitting vs. standing positions [34].

### Statistical analysis

The demographic and clinical characteristics of the participants were described using measures of central tendency and dispersion. Differences in GPS life-space measurements and trip frequency, duration, and mode between the Qstarz and the TicWatch models were compared using paired t-tests for normally distributed data, and the Wilcoxon signed-rank test for nonparametric data.

The relationship between the TicWatch S2 and ActiGraph for the time spent in sedentary and non-sedentary behavior (light and moderate/vigorous) was determined using Spearman’s rank correlation coefficient rho. The percentage agreement reported throughout the results section was calculated as (TicWatch/ActiGraph) x 100.

We constructed Bland–Altman plots [35, 36] to visualize the variability in step count recordings, compared to the participants’ self-reported step counts and the time spent on sedentary and non-sedentary behavior between the TicWatch and ActiGraph. We also plotted the mean activity counts per 60-second epoch length for six selected activities to investigate the extent of agreement between the devices. One way to assess the accuracy of the results is by analyzing the graphical representation of the mean error score and the 95% prediction intervals. Results that are more reliable will show a mean bias closer to zero and narrower 95% prediction intervals. Statistical analysis was conducted using SPSS (IBM SPSS Statistics Version 26). For all analyses, the significance level was set at α = 5%.

## Results

Data from 25 participants were used for Experiment 1, and 10 of those agreed to participate in Experiment 2. Participants’ characteristics are described in Table 1.

**Table 1:** Demographic and clinical characteristics of the study participants.

Participants’ characteristics	Experiment 1	Experiment 2
	Mean ± SD or N (%)	Mean ± SD or N (%)
	N = 25	N = 10
Age (years)	66.2 ±8.5	67.6 ± 6.3
Min-max	55–82	
Sex, N (%)		
Females	15 (60.0)	8 (80)
Males	10 (40.0)	2 (20)
BMI (Kg/m^2^)	27.5 ± 4.5	27.7 ± 3.2
Level of Education, N (%)		
Some post-secondary school	3 (12.0)	
Secondary school complete	5 (20.0)	
Post-secondary degree complete	17 (68.0)	
Have you had a fall in the past year? N (%)	6 (24.0)	4 (40.0)
How many falls? N (%)		
1	1 (4.0)	1 (10.0)
2 or more	5 (20.0)	3 (30.0)
Do you have difficulty with balance? N (%)		
yes	2 (8.0)	-
No	23 (92.0)	10 (100.0)
Daily number of medications, N (%)		
0	6 (24.0)	2 (20.0)
1	8 (32.0)	4 (40.0)
2	5 (20.0)	2 (20.0)
3 or more	6 (24.0)	2 (20.0)
Comorbidities, n (%)		
0	3 (12.0)	2 (20.0)
1	7 (28.0)	4 (40.0)
2	6 (24.0)	1 (10.0)
≥3	9 (36.0)	3 (30.0)
Preclinical Mobility Limitation*		
No mobility limitation	20 (80.0)	9 (90.0)
Preclinical mobility limitation	2 (8.0)	-
Minor mobility limitation	3 (12.0)	1 (10.0)

*Preclinical Mobility Limitation scale [8]

In Experiment 2, all participants consented to wear the thigh device. The majority (80%) expressed willingness to wear the device for a 10-day period, found the instructions easy to follow, and reported no impact on their daily routine. One participant experienced skin redness due to the adhesive patch. Among the adhesives tested (Cardinal Tegaderm, 3M Tegaderm, a white non-woven adhesive from 3M, and the Hypafix stretch non-woven), the majority preferred to use the Hypafix^TM^ Stretch Non-Woven Adhesive (BSN Medical) to affix the device to their thigh.

### Device performance

In Experiment 1, the analysis showed that all 25 TicWatch S2 devices had complete GPS data and the Qstarz had 22; thus, data from 22 participants are presented. The TicWatch S2 and ActiGraph devices had complete accelerometer data for 25 participants.

The average reported wear time on the diaries was 831.9 minutes (13:51:53) per day. The TicWatch S2 devices were worn on average for 810.9 minutes (13: 30:52) per day and the ActiGraph for 847.9 minutes (14:07:52) per day. The wear time percentage of agreement between the diaries and the devices was 93% for the TicWatch S2 and 96% for the ActiGraph, and between the TicWatch S2 and the ActiGraph was 95%.

On average, the TicWatch S2 collected GPS and sensor data for the first 621.0 minutes (10:21:00) and collected only sensor data for the final 200.0 minutes (3:20:00). The Qstarz device was able to collect GPS data for the entire day across all valid participants.

In Experiment 2, all 10 participants had complete GPS and thigh-worn ActiGraph data. The results of the battery life modes tested in Experiment 2, showed that, on average, the periodic fix collection was able to accommodate 13 hours and 23 minutes of GPS data, and the stay connected fix collection, 12 hours and 40 minutes. Our data showed a total of 14 days of GPS data collected during waking hours, gathered across 10 participants over the course of the study. Two of the days were observed to have significant GPS drift issues on the Qstarz and were removed from the analysis. All remaining days had at least 8 hours of wear time data collected and had captured at least one out-of-home trip event. Thus, a total of 12 valid days of GPS data were compared between the Qstarz and the TicWatch Pro 3 Ultra GPS.

The on-body detection of the TicWatch Pro 3 Ultra GPS registered an average 803.4 min (13:23:22) of use compared with 799.3 min (13:19:20) recorded by the participants. The mean percentage of agreement between the on-body detection and the wear time reported by the participants was 99.5%.

### GPS-related mobility measures

Table 2 shows a comparison of the GPS data between the Qstarz and the TicWatch models in both experiments. In experiment 1, participants completed an average of 9.1 (2.8) trips during the 3-day protocol as recorded by the Qstarz device. The TicWatch S2 recorded 6.2 (4.8) trips, and this difference was statistically significant (p = 0.001). The average trip duration recorded by the Qstarz was 179.5 ± 102.8 minutes (02:59:30), and by the TicWatch S2 was 211.6 ± 142.5 minutes (03:31:36), and no significant difference was observed (p = 0.091). The TicWatch Pro 3 Ultra GPS performed better than the S2 model in trip frequency measures and showed no statistically significant difference in trip duration and time spent in active and passive modes of transportation compared to the Qstarz. In addition, both TicWatch models performed similarly to the Qstarz logger in measures of life-space mobility (Table 2).

**Table 2:** Comparison of the mobility outcomes between the Qstarz GPS receiver and the different TicWatch models.

Variables	Experiment 1			Experiment 2		
	(N = 22)			(N = 10)		
	Qstarz	TicWatch S2	P-value	Qstarz	TicWatch Pro 3 Ultra GPS	P-value
	Mean	Mean		Mean	Mean	
	SD	SD		SD	SD	
**Trip Frequency and Mode**						
Trip Frequency (n)	9.1	6.2	^a^0.001	4.0	3.7	0.234
	2.8	4.8		2.8	2.0	
Trip Duration (min)	179.5	211.6	0.091	105.4	112.5	0.530
	102.8	142.5		84.5	77.3	
Passive Time (min)	-	-	-	26.9	23.5	0.398
				32.2	31.6	
Active Time (min)	-	-	-	78.5	88.9	0.374
				77.3	83.7	
**Life-space Mobility**						
Farthest distance from home (m)	1.8 × 10^4^	1.8 × 10^4^	0.545	4.6 × 10^4^	4.5 × 10^4^	0.099
	2.6 × 10^4^	2.6 × 10^4^		3.5 × 10^4^	3.5 × 10^4^	
Convex Hull						
Length (m)	4.1 × 10^4^	4.1 × 10^4^	0.053	1.4 × 10^4^	1.4 × 10^4^	0.269
	5.5 × 10^4^	5.5 × 10^4^		8.8 × 10^4^	8.7 × 10^4^	
Area (m)	1.3 × 10^8^	1.3 × 10^8^	.131	1.1 × 10^4^	1.1 × 10^4^	0.134
	3.0 × 10^8^	3.0 × 10^8^		1.2 × 10^4^	1.1 × 10^4^	

n = number; m = meters; SD = standard deviation

^a^Wilcoxon Signed Ranks test significant at 0.05 level

### Activity counts per minute and physical activity (PA) intensity

**Fig 2:**
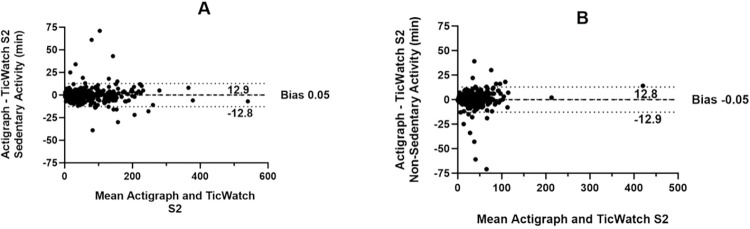
Bland-Altman plots illustrating A) the difference in the time spent in sedentary activity between the ActiGraph and TicWatch S2 and, B) the difference in the time spent in non-sedentary activity between the ActiGraph and TicWatch S2 Dashed lines = 95% limits of agreement (1.96 SDs of the mean difference); darker dashed line = mean difference or bias.

Table 3 presents the mean time in minutes spent in sedentary and non-sedentary behavior during the real-life mobility protocol, as measured by the ActiGraph and TicWatch S2 devices. Our analysis showed strong correlations between the two devices for both sedentary (r = 0.99, p < 0.001, see Table 3) and non-sedentary (r = 0.96, p <0 .001, see Table 3) behavior. In addition, the agreement rates between the TicWatch S2 and the ActiGraph algorithms were high for both sedentary (86.9%) and non-sedentary (83.7%) activities. The Bland-Altman plots shown in Fig 2A and 2B demonstrate that there was a high level of agreement between the two devices for both sedentary and non-sedentary behavior, as evidenced by the minimal bias and narrow 95% limits of agreement.

**Table 3:** Comparison of the time (min) spent in sedentary and non-sedentary (light, moderate/vigorous) behavior between the ActiGraph and TicWatch S2 during the real-life mobility protocol (N = 25).

Intensity Classification	ActiGraph	TicWatch S2	r	P-Value	Mean % Agreement
**Sedentary (min) Mean ± SD**	48.7 ± 16.3	48.6 ± 16.1	0.99	0.001	86.9
**Non-sedentary (min)** **Mean ± SD**	26.4 ± 20.9	26.5 ± 21.2	0.96	0.001	83.7

r = correlation coefficient

**Fig 3:**
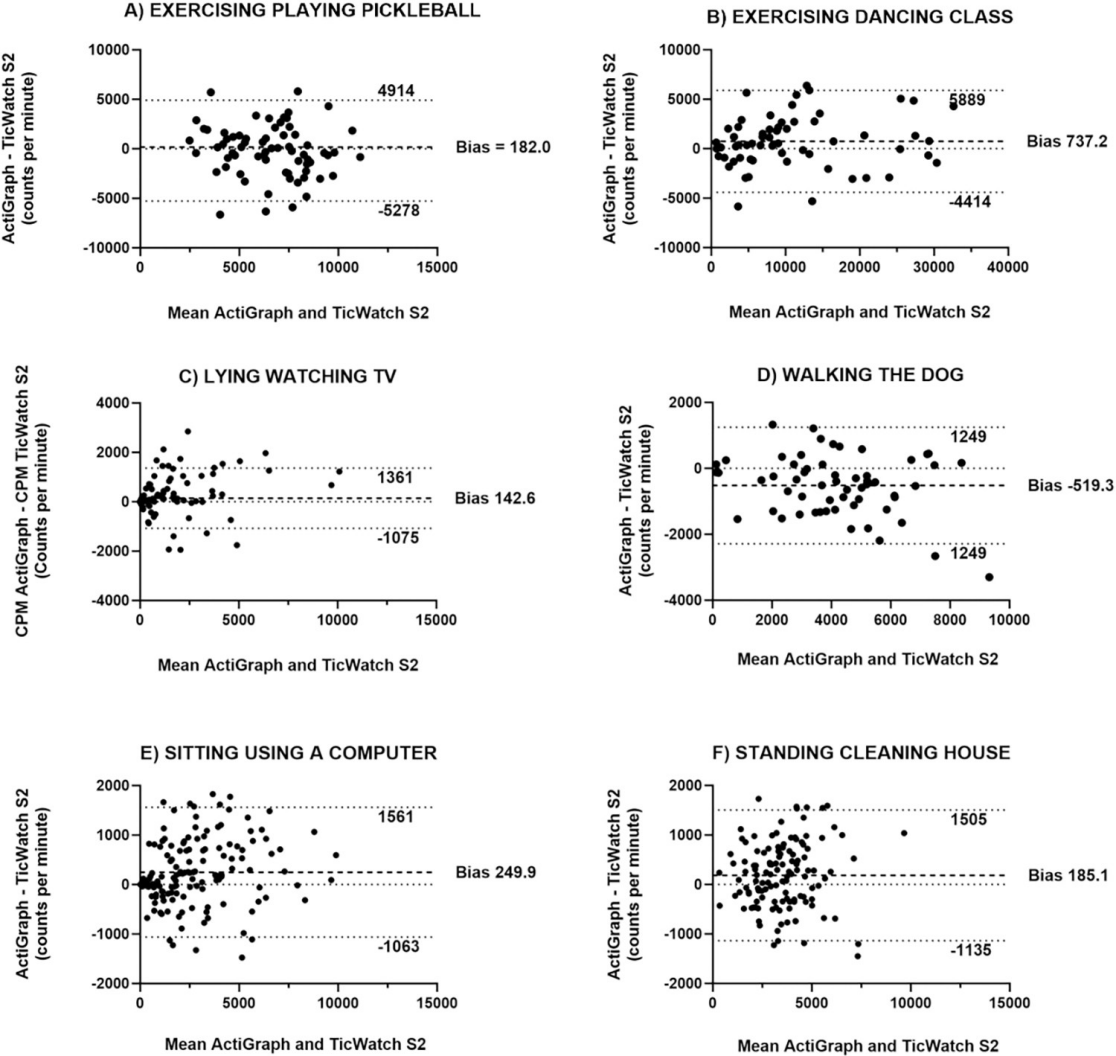
Bland-Altman plots illustrating the difference in the average counts per minute per 60-minute epoch length, between the ActiGraph and TicWatch S2 during the real-life mobility protocol activities reported by the participants. Dashed lines = 95% limits of agreement (1.96 SDs of the mean difference); darker dashed lines mean difference or bias.

The Bland-Altman plots in Fig 3A–3F illustrate a comparison of the average counts per minute per epoch length of 60 seconds for some of the activities reported by six participants, using both ActiGraph and TicWatch devices. The plots show that there is no systematic error in the comparison between the two devices and that there is a small bias observed for most activities. While a few observations fell outside the 95% limits of agreement, the majority of observations were within this range, indicating a good level of agreement between the TicWatch device compared to the medical grade ActiGraph.

### Step count and body posture

**Fig 4:**
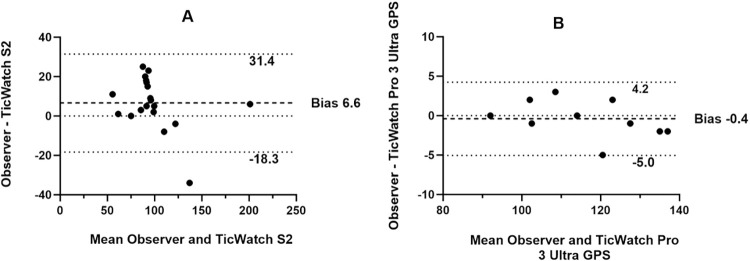
Bland-Altman plots illustrating A) the difference between the self-reported step count and the TicWatch S2 (N = 21), and B) difference between the self-reported step count and TicWatch Pro 3 Ultra GPS (N = 10) during the step count task. Dashed lines = 95% limits of agreement (1.96 SDs of the mean difference); darker dashed line mean difference or bias.

In the step count analysis, data from 21 of the 25 participants were used, as the TicWatch S2 step counter algorithms failed to detect any steps for four participants during the test period. For experiment 2, data from all 10 participants are presented. Bland-Altman plots (Fig 4) indicate that the TicWatch Pro 3 Ultra GPS showed narrower limits of agreement, closer observations to zero, and a smaller bias of 0.4 steps, in comparison to the TicWatch S2, which overestimated the number of steps by 6.6 steps.

The algorithm used by the thigh-worn ActiGraph device exhibited a high degree of accuracy in classifying body postures, with a 97% accuracy rate in correctly identifying sitting and lying postures and a 90% accuracy rate in identifying standing postures.

## Discussion

Our findings revealed that the TicWatch Pro 3 Ultra GPS outperformed the S2 model and was comparable to the Qstarz in measuring life-space mobility, trip frequency, duration, and mode of transportation. The device’s long-lasting battery life of over 10 hours per day makes it particularly suitable for research purposes, given the recommended daily wear time of 10 hours [27]. In terms of accelerometer data, the TicWatch S2 was found to be comparable to the counts per minute generated by the ActiGraph. We also showed that the thigh-worn ActiGraph has high accuracy for detecting body posture. These findings support the validity of our multi-sensor approach for real-world mobility monitoring. Furthermore, given that wrist-worn devices are increasingly popular among older adults, the TicWatch’s accurate measurement capabilities and user-friendly design make it a promising tool for monitoring mobility in large cohort studies.

We chose to derive key indicators of mobility using the GPS data as informed by prior studies [14, 30–32]. Specifically, we identified life-space extent (maximum distance from home and minimal convex hull), trip frequency, trip duration, and travel mode. These measures were chosen as they are frequently used in prior studies on older adult mobility and together provide information on the space, time, movement scale, and attribute characteristics of a person’s mobility [14, 21]. Despite the relatively small absolute difference between the TicWatch S2 in Experiment 1 and the standalone Qstarz for capturing trip frequency and duration, this difference was statistically significant for trip frequency. However, for all reported measures in Experiment 2 with the TicWatch Pro 3 Ultra GPS model, there were no significant differences between the measures obtained from each device, which we attribute to the newer model’s improved hardware specifications. Thus, the increased frequency of data collection and prolonged battery life of the TicWatch Pro 3 Ultra GPS device likely contributed to improving the accuracy of trip frequency detection. Furthermore, we observed that the trip duration accuracy also improved in Experiment 2 with the newer hardware. Although the Qstarz and TicWatch devices showed comparable accuracy for navigation data overall in both experiments, some variation was still observed. This can be attributed to measurement errors in the TicWatch device, which was found to be more sensitive to poor satellite signal reception when determining location accuracy compared to Qstarz. Moreover, the difference in data collection frequency between the devices (i.e., 5 seconds vs. 20 seconds in Experiment 2) sometimes caused the TicWatch’s trip algorithm to group together long and short trips, leading to further discrepancies.

Of note, passive and active time, which represent segments of time spent using active (i.e., walking, biking) or passive (i.e., car and bus) transport modes, were also comparable between the TicWatch devices and Qstarz. As this measure is based on speeds derived from the watch’s GPS data, it provides further validation of the data quality from the watch. Finally, life-space mobility measures, including the farthest distance from home and the minimum convex hull, were derived. Interestingly, these measures performed quite well on the TicWatch in both experiments despite the difference in GPS quality. Thus, it appears that both measures do not require high-frequency GPS data to be accurately derived, which makes intuitive sense as they rely on the aggregation of the full underlying dataset. Having GPS points every 5 seconds compared to every 20 seconds did not substantially affect the measured farthest distance, which makes sense as the participant would need to travel a significant distance in a very short period to cause a major discrepancy. Likewise, minimum convex hulls only represent the outer spatial bounds of the data, and thus were not as sensitive to missing detail within the GPS data. This finding suggests that these measures, and possibly other aggregate measures of the global dataset, could perform well regardless of the quality of the GPS device being used, which has potential cost savings implications for future studies. Although the measures presented here serve as a proof-of-concept for the TicWatch as a GPS collection device, future work can explore deriving additional mobility measures using the data to provide a more comprehensive understanding of the dimensions that shape mobility in healthy aging.

Our analysis of step counts indicated that the TicWatch Pro 3 Ultra GPS model consistently matched the participants’ self-reported step counts during their walking task (Fig 4B). It is important to note that, in Experiment 1, we utilized the step counter algorithms, which recorded the total number of steps taken over a given period. This sensor periodically calculates the number of steps taken since its last reading and adds them to the overall count. As a result, the recorded steps included some that were not part of the test, causing the TicWatch S2 to overestimate the number of steps (mean error = 6.6 steps) compared to the self-reported step count. It should be noted that this type of sensor is designed to continuously track steps, even when the watch app is in suspended mode. In Experiment 2, we selected the step detector sensor, which records each step as it occurs in real-time. It generates an event each time it detects a step, but it does not keep track of the total number of steps taken. This sensor is better suited for a test conducted under controlled conditions because it records each step in real-time, allowing us to count the number of steps between the beginning and end of the step task with greater accuracy. This likely explains why the Pro 3 Ultra GPS model shows closer agreement with participants’ self-reported data compared to the S2 model.

Wrist-worn devices are popular and convenient for tracking physical activity levels, but research has shown that they can be challenging during real-life conditions due to factors such as excess arm movements, which can induce false step detection, or activities in which the wrist may not move, such as pushing a grocery cart or a stroller, which can lead to an underestimation of step counts [37–39]. This can result in inaccurate readings and potentially misinform the user about their activity levels. To improve accuracy, more advanced approaches such as machine learning and GPS can be employed to enhance step count accuracy further [40, 41]. Combining GPS and accelerometry could also improve step detection accuracy by compensating for the limitations of each individual technology. GPS is highly effective at providing accurate distance and location information but can be subject to signal loss or interference in certain environments. Accelerometers, on the other hand, are highly sensitive to movement and can detect even small changes in motion but cannot accurately determine distance or location. Nevertheless, the findings of this study support the performance of the wrist-worn TicWatch models for assessing step count under controlled conditions. However, further investigation is needed to establish the accuracy of the TicWatch Pro 3 Ultra GPS in measuring step counts in unconstrained environments.

Research has shown that sedentary behavior can have serious negative effects on health, including an increased risk of obesity, cardiovascular disease, cancer, and mortality, especially among older adults [42–44]. Using accelerometry, sedentary behavior is measured by tracking body posture or the time an individual spends sitting or lying down, as compared to standing or walking, or by estimating the PA intensity (sedentary, light, moderate/vigorous). Accurately measuring sedentary behavior can be challenging, requiring devices placed at more than one anatomical location [45, 46]. However, using multiple devices can lead to reduced participant compliance, necessitating reliance on a single device. The ActiGraph’s thigh-worn inclinometer algorithm is one such device that uses a movement threshold and thigh angular orientation to distinguish between lying and sitting or sedentary behaviour, from standing and stepping [34]. Compared to other algorithms such as the activPAL, the ActiGraph’s thigh angular parameter improves the classification of sitting posture, even when the participant has their legs crossed or stretched, in both laboratory and real-life conditions [47–49]. The findings of Steeves et al. [48] support our study, as they reported that the ActiGraph thigh algorithm accurately classified 100% of all sitting activities and 83% of all standing activities in laboratory settings. In our study, we found that the thigh-worn algorithm correctly classified sedentary behavior (sitting and lying) 97% of the time, and standing 90% of the time, during our 5-minute sitting and standing tasks [48]. Results from our feasibility questionnaire indicated that participants were willing to wear the ActiGraph thigh device continuously for more than three days in real-world conditions and provided valuable insights for determining the most appropriate adhesive patch, the Hypafix^TM^. These results will help us implement the use of the thigh-worn ActiGraph with confidence in a large cohort study of older adults, contributing to a better understanding of the relationship between sedentary behavior and health outcomes.

We also evaluated the TicWatch as a measure of physical activity (PA) intensity and compared its counts per minute (CPMs) with the gold-standard ActiGraph device. Our analysis revealed a strong correlation between the time spent in sedentary and non-sedentary (r≥.96) behavior (light, moderate/vigorous), with a good agreement range of 83.7% to 86.9%, supporting that the accelerometer output from the devices is similar. We chose Montoye’s [29] cut-offs due to their accuracy (71%) in estimating physical activity (PA) intensity in real-life mobility settings, comparable to cut-offs obtained through machine learning (58–61% accuracy) [50]. To address the issue of accuracy in estimating moderate and vigorous physical activity levels, we combined the light and moderate/vigorous activity categories. This decision was based on studies that reported that wrist-worn devices are comparable to waist-worn devices in estimating sedentary behavior, but lack accuracy in estimating light and moderate/vigorous activity due to a combination of factors like placement and orientation of the device, type of physical activity performed and errors due to sporadic wrist movements [29, 51]. To gain further insights into the activity counts per minute, we plotted the mean activity counts per 60-second epoch length for various activities, including exercising, sitting, lying, and walking, as reported by the participants (Fig 2A–2F). We observed no systematic differences between devices. Although the bias was relatively small, we observed some variability in the results, which may have been influenced by issues related to synchronization due to differences in the internal clock or timekeeping mechanism of the accelerometers. It is important to note that even if the accelerometers were set to the same time zone, their internal clocks may have drifted or had varying levels of precision, leading to differences in the recorded time between the accelerometers. Additionally, since the participants wore two devices simultaneously on the same wrist, it is possible that one of the devices was more loosely fitted than the other, resulting in some degree of variation in the readings. Despite this limitation, we demonstrated that the accelerometer from the TicWatch has the potential to be further explored in determining PA intensities.

Our study’s strength lies in the validation of a commercially available smartwatch for collecting movement and navigation data to comprehensively monitor real-world mobility in older adults. Equipped with our custom applications Ivy and Clover, the TicWatch collects and stores raw sensor measurements (accelerometer, gyroscope, heart rate and ambient light), step counts, activity type predictions, and location (GPS) data, thereby avoiding the need for a second standalone GPS device. Most commercially available devices only store outcome measures and do not store the raw data. The ability of the TicWatch to collect raw sensor measurements allows for much more in depth analyses with algorithms that are tuned specifically for older adults. However, some limitations should be considered. Due to the COVID-19 pandemic, the experiments were conducted entirely remotely, with participants completing standardized tasks at home. This may have affected the accuracy and generalizability of the results, as participants’ daily routines and mobility patterns may have differed from those under normal circumstances. Additionally, since the participants completed the tasks and activity logs without our supervision, there is a possibility that some reported activities were not accurately recorded.

## Conclusion

We have demonstrated that our multi-sensor approach to mobility monitoring shows promise for obtaining both traditional accelerometer-derived movement data and for capturing trip and life-space data only obtainable via GPS. Specifically, the TicWatch models investigated in this study are valid devices for capturing GPS and raw accelerometer data, making them suitable for use in assessing the real-world mobility of older adults and furthering our understanding of early mobility decline. These findings have significant implications for the development of larger cohort studies and for the field of wearable technology in Canada and beyond.
